# Emotional Meaning in Context in Relation to Hypomanic Personality Traits: An ERP Study

**DOI:** 10.1371/journal.pone.0138877

**Published:** 2015-09-22

**Authors:** Sarah Terrien, Pamela Gobin, Alexandre Coutté, Flavien Thuaire, Galina Iakimova, Pascale Mazzola-Pomietto, Chrystel Besche-Richard

**Affiliations:** 1 Laboratoire Cognition, Santé, Socialisation, C2S, EA 6291, Université de Reims Champagne-Ardenne, Reims, France; 2 Laboratoire d'Anthropologie et de Psychologie Cognitive et Sociale, LAPCOS, EA 7278, Université de Nice-Sophia Antipolis, Nice, France; 3 Institut des Neurosciences de la Timone, CNRS UMR 7289, Marseille, France; 4 Institut Universitaire de France, Paris, France; University Zurich, SWITZERLAND

## Abstract

The ability to integrate contextual information is important for the comprehension of emotional and social situations. While some studies have shown that emotional processes and social cognition are impaired in people with hypomanic personality trait, no results have been reported concerning the neurophysiological processes mediating the processing of emotional information during the integration of contextual social information in this population. We therefore chose to conduct an ERP study dealing with the integration of emotional information in a population with hypomanic personality trait. Healthy participants were evaluated using the Hypomanic Personality Scale (HPS), and ERPs were recorded during a linguistic task in which participants silently read sentence pairs describing short social situations. The first sentence implicitly conveyed the positive or negative emotional state of a character. The second sentence was emotionally congruent or incongruent with the first sentence. We analyzed the difference in the modulation of two components (N400 and LPC) in response to the emotional word present at the end of the “target” sentences as a function of the HPS score and the emotional valence of the context. Our results showed a possible modulation of the N400 component in response to both positive and negative context among the participants who scored high on the Mood Volatility subscale of the Hypomanic Personality Scale. These results seem to indicate that the participants with hypomanic personality traits exhibited specificities in the integration of emotions at the level of the early-mobilized neurocognitive processes (N400). Participants with hypomanic personality traits found it difficult to integrate negative emotional contexts, while simultaneously exhibiting an enhanced integration of positive emotional contexts.

## Introduction

Hypomanic personality (HP) describes people who are cheerful, optimistic, extraverted, self-confident and energetic, although sometimes also irritable, rude, reckless and irresponsible [1–3]. Hypomanic personality traits can be assessed using the Hypomanic Personality Scale (HPS) [2] which contains three factors: Social Vitality, Mood Volatility and Excitement [4]. The Social Vitality factor refers to social potency and vivaciousness. The Mood Volatility factor explores unpredictable mood states and hypomanic cognition. The Excitement factor refers to energetic, extremely cheerful moods. High scores on the HPS scale have been shown to predict the onset of manic or hypomanic episodes over a 13-year period [5]. Indeed, hypomanic personality is associated with an increased risk of subsequently developing bipolar disorders (BD) [6].

Beyond the common clinical features, there are similarities in certain areas of cognitive functioning in BD and HP, and in particular at the level of emotional processes and social cognition. BD patients have an impaired ability to process social situations that involve emotional processing requiring the recognition of emotional facial expressions [7–9] as well as an impaired affective and cognitive Theory of Mind (ToM) [7, 10, 11]. A recent study has shown that, unlike women with HP traits, men with such traits also present a deficit in cognitive ToM [12]. Furthermore, a recent fMRI study of HP individuals has shown that high HP is associated with inefficient regulation of emotions at the neural level (e.g., [13]) and that this might be considered to be a cognitive vulnerability marker for BD. Based on the observation of an increase in positive emotions in response to positive, negative and neutral film clips [14], some authors have described the regulation of emotions in HP in terms of a positive interpretation bias [14]. This emotional style, involving a higher level of positive emotions in response to emotional stimuli, has been observed in a survey of people with mania and with tendencies toward mania [15]. Therefore, it appears that, in the same way as in BD, individuals with HP might also exhibit a positive bias in response to emotional stimuli. The present study explored this question by focusing on on-line emotional context processing.

The ability to integrate contextual information is important for the comprehension of emotional and social information [16] and can be explored, during on-line language comprehension, by means of Event-Related Potentials (ERP). N400 is a negative event-related potential which appears 400 ms after the presentation of a target. The amplitude of the N400 component is more negative when the target is incongruent with the context than when it is congruent, especially in semantic contexts [17]. Another late component has also been studied in ERP studies dealing with contextual integration: the Late Potential Component (LPC). The LPC is a positive component which occurs 600 ms after the stimulus. It has classically been associated with syntactic processing and its amplitude increases when a syntactic violation occurs (e.g., [18]). Other authors have suggested that this component could be associated with the general updating of the context [19] or with the attentional analysis of the overall meaning of a sentence [20].

The use of semantic context can be explored by manipulating the semantic context, irrespective of whether this context is a word (e.g., classical semantic priming paradigm), a whole sentence, or a corpus of sentences. BD individuals are thought to exhibit difficulties in semantic processing, as has been shown by Andreou et al. [21] who reported that remitted bipolar patients exhibited a reduced semantic priming effect in a classical SP paradigm.The authors suggested that semantic processing may be abnormal in bipolar disorder patients Abnormalities in the use of semantic context can also be explored through ERP studies such as that conducted by Ryu et al. [22]. This study used a word-matching task in which participants had to decide whether or not the prime word semantically matched the target word. The results revealed that manic patients exhibited an increased N400 amplitude on semantically congruent words and a reduced N400 amplitude on semantically incongruent words compared to healthy participants. Based on these results, the authors concluded that these results were the consequence of a disruption of the working memory needed to maintain contextual integration [22]. In order to explore the ERPs of bipolar patients compared to those of healthy participants in natural speech conditions, Cermolacce et al. (2014) conducted a study using sentences that ended either with an expected semantically congruent word (e.g., “The car was driven by a driver”) or with an unexpected incongruent word (e.g., “I went to see the boats on the door”) [23]. The participants’ task was to judge whether the last word of each sentence was congruent or not. This study revealed a preserved N400 component in BD manic patients when compared to healthy participants. However, the behavioral performances of the BD manic patients were impaired compared to those of the healthy participants: the patients made more errors on both the congruent and incongruent endings and their reaction times on the congruent endings were longer than those of the healthy participants [23]. The differences observed between the two ERP studies could be due to the nature of the stimuli and to the characteristics of the clinical patients. Indeed, Cermolacce et al. [23] used a more ecological task than Ryu et al. [22] (word matching task *vs*. listening to semantically congruent and incongruent sentences). Furthermore, the patients in Cermolacce et al.’s study were older, less educated, took more antipsychotics and had higher scores on the Brief Psychiatric Rating Scale than Ryu et al.’s patients. Concerning the LPC, Cermolacce et al. observed similar patterns of amplitude in both the manic BD patients and the healthy participants, but with a delayed latency effect in the manic patients [23]. According to the authors, the delayed LPC latency may illustrate a disturbance in very high level integration processes in the BD manic population.

Given that HP present score reflects a vulnerability to BD and that BD patients present abnormalities in their use of context, we chose, in our study, to investigate possible abnormalities in the use of context in HP. More specifically, given the bias toward positive interpretation in HP, we investigated possible abnormalities by taking negative emotional context in HP into account. More specifically, given the bias toward positive interpretation in HP, we investigated possible abnormalities in taking account of negative emotional context in HP. The aim of the present study was to investigate whether the Mood Volatility dimension of the HPS and the valence of the emotional context would influence the integration of an emotional adjective. We used an ERP study that focused on the modulation of two components (N400 and LPC) as a function of the HPS score and the emotional valence of the context.

To create an emotional context, the participants had to read sentences referring to the emotional states (positive or negative) of different persons. Each of these sentences was followed by either an emotionally congruent or incongruent target sentence. In order to investigate the integration of emotional context in a more lifelike, “ecological” condition, we decided to use a method taken from the passive reading task. Indeed, our task was inspired by Holt et al.’s study [24] in which the authors interpreted the results obtained during a passive reading task as indicating “naturalistic online comprehension”. Given that a high score on the Mood Volatility subscale of the HPS is considered to be a better predictor of the development of BD and impairment in social settings than the other two HPS subscales [4], we hypothesized that healthy participants with high scores on the Mood Volatility subscale would find it more difficult to process emotional information during the integration of contextual social information than participants with low scores on this subscale. We chose to study the integration of emotional information on the basis of N400 and LPC measurements. We expected the participants who scored low on the Mood Volatility subscale to exhibit larger N400 and LPC amplitudes during the reading of the incongruent target sentences than of the congruent target sentences, independently of the valence of the emotional context. Given that Gruber et al. [14] suggested that individuals with a high level of hypomanic personality traits possess a bias toward positive interpretations, we hypothesized that the valence of the context would influence the processing of context integration and that participants with high scores on the Mood Volatility subscale would find it easier to integrate positive rather than negative contexts. We therefore assumed that the modulation of the N400 and LPC effects would also depend on the valence of the emotional context sentence. Furthermore, it has been shown that the LPC and N400 do not reflect the same stage of cognitive processing: compared to the N400 component, LPC tends more to reflect an aspect of the cognitive or executive control of language[25]. Moreover, a recent study has shown that the emergence of the automatic processes involved in the integration of multimodal emotional information is delayed in BD patients [26]. In line with Gruber et al.’s results [14] (suggesting a positive interpretation bias in hypomanic personality trait) and those obtained by Van Rheenen et al. (suggesting a dysfunction in the automatic process of emotional integration in BD), we expected to observe: 1) in the emotionally incongruent condition, a reduced N400 amplitude only in response to negative context sentences in participants who scored high on the Mood Volatility subscale of the HPS; 2) in both the emotionally congruent and incongruent conditions, a preserved LPC amplitude in participants who scored high on the Mood Volatility subscale of the HPS.

## Method

### Participants

Seventy two participants (57 women and 15 men; mean age = 24.41 years ±6.37; mean years of education = 14.79 ±2.83) from the University of Reims Champagne-Ardenne, the University of Nice Sophia Antipolis and the University of Aix-Marseille were included in the experiment. They were recruited by means of information letters. All participants were French native speakers, had normal or corrected-to-normal vision, had no past or present history of reading disabilities, and no neurological or psychiatric disorders. Furthermore, none of them reported taking any psychotropic medications. All the participants completed the HPS [2]. This self-report scale consists of 48 true-false items relating to hypomanic personality features. The HPS score is subdivided into 3 factors: Social Vitality, Mood Volatility and Excitement [4]. The participants also completed the Mill-Hill vocabulary test [27] which assesses stored verbal knowledge and its retrieval. Our subgroup of participants was divided *a posteriori* into a Low-Mood Volatility HPS group (l-M-HPS; n = 34) and High-Mood Volatility HPS group (h-M-HPS; n = 33) on the basis of the median value (i.e., 6) of the HPS Mood Volatility factor. Participants with an HPS Mood Volatility score equal to the median were excluded (n = 5).

### Ethics Statement

This study was designed in accordance with the Declaration of Helsinki. The "Comité de Protection des Personnes—Est III" of the University hospital of Nancy approved the protocol (Permit number: 2013-A000493-42). The anonymity of all the participants was ensured and they gave their written informed consent prior to the study.

### Stimuli and design

The stimuli consisted of 66 short written scenarios, all of which consisted of two sentences: a context sentence followed by a target sentence. The context sentence (6–12 words) implicitly conveyed either the positive or negative emotional states of a person (e.g. negative: "The girl has lost her best friend"). The target sentence (3–5 words) ended in an emotional adjective. A distinction was made between two types of "target sentence": 1) 33 target sentences ended with an adjective that was congruent with the character's emotional state (e.g. "The girl is sad") as implicitly described in the context sentence; 2) 33 target sentences ended with an adjective that was incongruent with the character's emotional state (e.g. "The girl is happy") as implicitly described in the context sentence. This design resulted in four experimental conditions: 1) Negative context—Emotionally Incongruent target sentence; 2) Negative context—Emotionally Congruent target sentence; 3) Positive context—Emotionally Incongruent target sentence; 4) Positive context—Emotionally Congruent target sentence. Additional filler sentences were included: 22 target sentences were "neutral" with regard to the contextually implied emotional state of the character (e.g. "The girl is seated”).

During a series of pre-tests, all the emotional adjectives used in the target sentences had been evaluated by 204 students to ensure that they had either a positive or a negative valence. The items were judged on a 7-point Likert scale ranging from -3 for negative to 3 for positive items, respectively. Each of the selected negative and positive items had a score of less than -1.5 or more than 1.6, respectively. The emotional valence of the context was evaluated by 2 independent experts.

**Fig 1:**
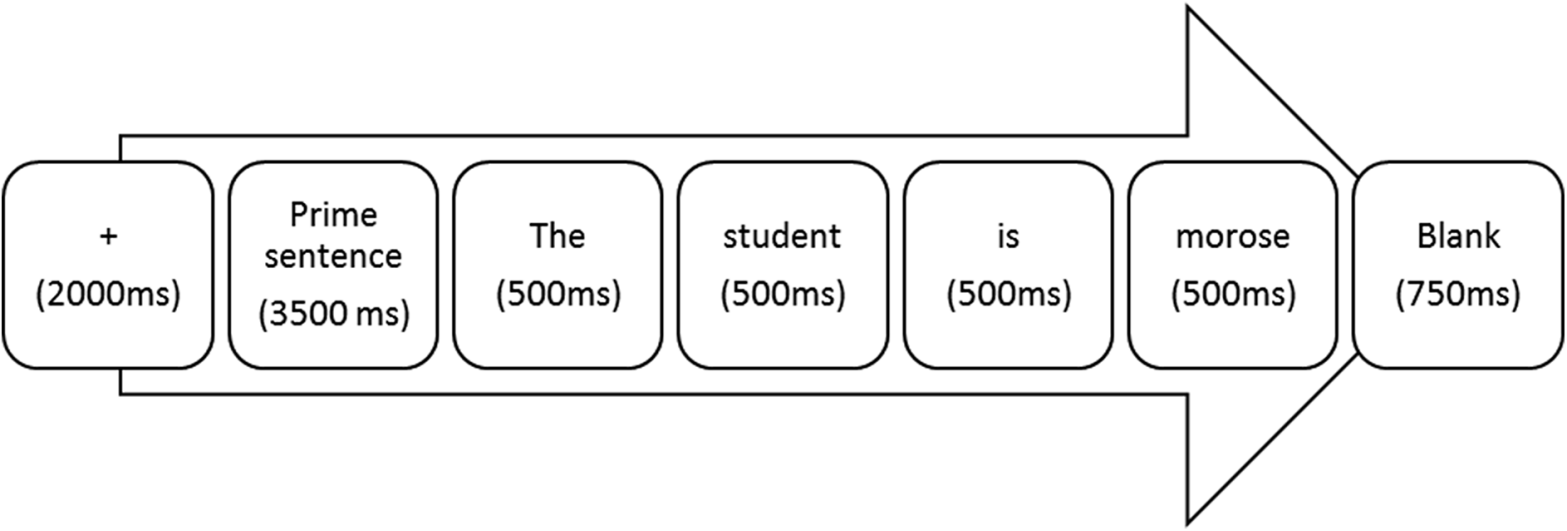
Trial procedure. Each trial had the following structure: a fixation point was presented in the center of the screen (2000 ms), followed by a context sentence (3500 ms) and a target sentence, with each word being presented sequentially (500 ms for each word). Each trial was followed by a black screen which was displayed for 750 ms.

The stimuli were presented in the visual modality in a fixed pseudorandom order. Each trial had the following structure: a fixation point was presented in the center of the screen (2000 ms), followed by a context sentence (3500 ms) and a target sentence, with each word being presented sequentially (500 ms for each word). Each trial was followed by a black screen which was displayed for 750 ms (see Fig 1). The inter-trial interval was 6750 ms. The sentences were presented with white characters on a black background, using E-Prime software (Psychology Software Tools, Pittsburgh, PA). The participants' were asked to complete a passive reading task.

### Electro-Encephalographic recording

Electro-Encephalographic activity (EEG) was recorded using a 32-channel Electrocap (BrainAmp, BrainProducts, Germany) arranged on the scalp according to the international 10–20 system. The reference electrode was positioned at an equal distance from Fz and Cz and the ground electrode was located between Fz and the pre-frontal sites. The electrode impedance was held below 5 kΩ. The amplification gain was 1000. To record the electro-oculogram, two electrodes derived from FP1 and FP2 were placed below the right eye and on the external canthus of the left eye and set to an amplification gain of 500. The EEG signal was digitized online at a sampling rate of 250 Hz, with bandpass half-amplitude cutoffs of .1 to 100 Hz, and was filtered using a 30 Hz low-pass filter. Before segmentation, all the electrodes were re-referenced offline to the digital average of the activity at the left and right mastoids. The EEG was segmented 200 ms before target onset and 1500 ms after target onset. Baseline correction was performed for the 200-ms epoch before target onset. Eye blinks were checked for using the Gratton and Coles method by means of an automated analysis performed by BrainVision Analyzer. Artifacts were also checked for by means of an automated analysis performed by BrainVision Analyzer. The following artifact rejection criteria were applied for the period 200 ms before to 200 ms after each segment: (1) maximum permitted voltage of 50 μV/ms, (2) maximum absolute difference of 100 μV for a 200-ms interval and (3) minimum required activity in a 100-ms interval of 0.5 μV. A visual inspection was also performed to check for blinks and other artifacts. Only participants for whom at least 50% of segments were retained were included in the analyses. Artifact-free EEG trials were averaged separately over a 1.7-second time window for each condition. The mean number and range of trials used for each average were equivalent: Negative context—Emotionally Incongruent target sentence (9.84 trials ± 1.47, 5–11), Negative context—Emotionally Congruent target sentence (10.48 trials ± 1.30, 7–11), Positive context—Emotionally Incongruent target sentence (10.09 trials ± 1.32, 6–11), Positive context—Emotionally Congruent target sentence (10.18 trials ± 1.79, 5–11).

### Data analysis

**Fig 2:**
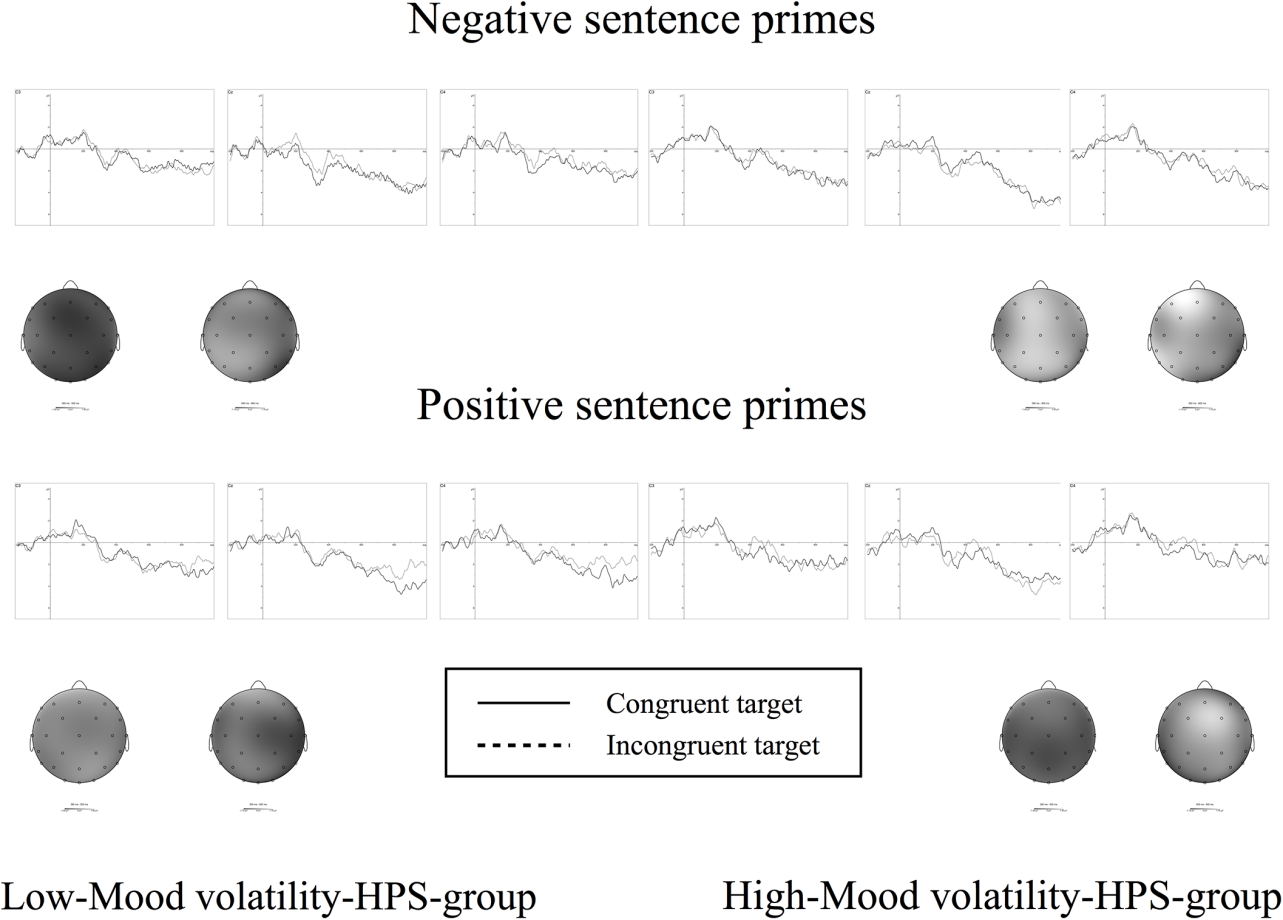
ERPs for the three central electrodes and topographical representations for the Low Mood Volatility-HPS and the High Mood Volatility-HPS as a function of type of Emotional valence and Congruency.

Statistical analyses were conducted using R software. To analyze the demographic and clinical data, we used two tailed *t-tests* and χ*²* when appropriate. ERPs were analyzed for the 9 electrodes placed on and around the medial line (Fz, Pz, Cz, F3, F4, C3, C4, P3, P4). The mean ERP amplitudes were submitted to a 4-way ANCOVA with one between-subject factor Group (Low Mood Volatility-HPS *vs*. High Mood Volatility-HPS), three within-subjects factors: "Congruency", which refers to the emotional congruency between the context and the target sentences (2: congruent *vs*. incongruent) x Emotional valence of the context sentence and referred to as “Emotional valence” here (2: positive, negative) x Electrode factor (9: F3, Fz, F4, C3, Cz, C4, P3, Pz, P4), and two covariates factors (Age and Education Level). Effects or interactions with the electrode factor were not described or discussed. The Greenhouse-Geisser correction (1959) was applied to correct violations of the assumption of sphericity when appropriate. Furthermore, for the 3-way interaction, pair-wise multiple comparisons (Tukey test) were run and the *p-values* are reported for significant comparisons. Based on a visual inspection and peak detection performed by Brain Analyzer, we focused our analysis of mean amplitude on two temporal windows: 380–500 ms for N400 and 500–900 ms for LPC (see Fig 2).

## Results

### Between-groups Comparisons

The Low and High Mood Volatility groups did not significantly differ with respect to verbal abilities. As expected, the two groups differed significantly in terms of HPS total score and HPS factor scores. The two groups also differed significantly at the level of age and years of education (see Table 1).

**Table 1:** Demographics and HPS characteristics for the Low and High Mood Volatility HPS participants included in the ERP analysis.

	Low Mood Volatility-HPS (n = 34)	High Mood Volatility-HPS (n = 33)	p-value
**Female/Male**	27/7	26/7	p = 0.95
**Age**	27.06	20.69	p< .001
**Years of education**	16.24	13.25	p< .001
**Mill Hill**	35.47	33.94	p = .056
**HPS-Total score**	6.94	22.73	p< .001
**HPS-Mood Volatility score**	2.35	10.82	p< .001
**HPS-Excitement score**	0.74	3.31	p< .001
**HPS-Social Vitality score**	3.85	8.81	p< .001

### ERP results

#### N400 component

ANCOVA revealed a significant Group x Congruency x Emotional valence interaction, *F*(1, 65) = 4.88, *p* = .031, *η* ^*2*^_*g*_ = .010. N400 mean amplitude for negative sentences in the Low-Mood Volatility HPS group was larger for emotionally incongruent (0.420 μV) than for emotionally congruent (1.187 μV) words (p < .01). By contrast, this effect was reversed in the High-Mood Volatility HPS group (Congruent: 0.927 μV *vs*. Incongruent: 1.291 μV) even if it was no significant according to post-hoc test (p > .10). As far as positive sentences, in the Low-Mood Volatility HPS group, N400 mean amplitude was slightly more negative for emotionally incongruent (1.003 μV) than for congruent words (1.052 μV). This difference was greater for the High-Mood Volatility HPS group (Incongruent: 0.470 μV *vs*. Congruent: 1.034 μV). However, none of these differences was significant according to post-hoc test (p > .10).

No other effect was significant (all *p*s > .05).

#### Late Positive Component

Results of ANCOVA showed no significant effect on the LPC mean amplitude (all *p*s > .05).

## Discussion

The aim of the present study was to investigate whether the Mood Volatility dimension of the HPS and the valence of the emotional context would influence the integration of an emotional adjective occurring at the end of a "target" sentence. So, we conducted an ERP study focusing on the modulation of two components (N400 and LPC) in response to the emotional word present at the end of the "target" sentences as a function of the HPS score and the emotional valence of the context.

### N400

For this time window, the integration of emotional valence during context processing seems to be influenced by the mood and the type of context. Indeed, we found a Group x Emotional Valence x Congruency interaction for this time window which is consistent with our hypothesis (planned comparisons were not performed because there was only one degree of freedom). As far as the negative context sentences are concerned, the Low-Mood Volatility HPS group exhibited a larger N400 for incongruent stimuli than for congruent stimuli, thus indicating that negative emotional contexts are integrated well. Indeed, classically, the amplitude of the N400 component is more negative when the target is incongruent with the context than when it is congruent [17]. The mean data indicated that the reverse pattern of N400 modulation was observed for the High-Mood Volatility HPS group, with a larger N400 amplitude in the congruent than in the incongruent condition. This N400 modulation is consistent with the results obtained in manic patients with an increase in N400 amplitude for semantically congruent words and a reduction of N400 amplitude for semantically incongruent words compared to healthy participants [22]. The observations concerning the High-Mood Volatility HPS participants can be explained, in line with Ryu et al.’s conclusions that this N400 modulation may reflect the consequence of a disruption of the working memory needed in order to maintain contextual integration [22], by the fact that the negative context sentences were incongruent with the participants' mood state. Indeed, the items included in this factor referred to a positively valenced mood (e.g., "I frequently get into moods where I feel very speeded-up and irritable", "I very frequently get into moods where I wish I could be everywhere and do everything"). Individuals with hypomanic personality might therefore have experienced a positive emotional state. This mismatch would lead to attention being withdrawn from the negative context sentence, with information not relevant to their emotional state being ignored in order to allow the individual to maintain his or her positive mood. Thus, when a target word was presented, the participants might have found it difficult to refer to the emotional context sentence and might therefore not have detected the incongruity between these two sentences correctly.

Concerning the positive context sentences, the mean data indicated that the High-Mood Volatility HPS participants seemed to exhibit a larger N400 amplitude difference between the incongruent and the congruent stimuli than the Low-Mood Volatility-HPS participants. This result could reflect the fact that the High-Mood Volatility HPS participants detected incongruities between positive contexts and negative targets better than the Low-Mood Volatility HPS participants. This observation can be explained by the fact that the positive context sentence was congruent with the mood state of the participants who scored high on the Mood Volatility subscale of the HPS. As we have seen above, individuals with hypomanic personality have a positive mood and the feeling they experience when reading positive sentences could be enhanced by their positive mood state. Thus, during the reading of the negative target sentence, the detection of emotional incongruence would be enhanced.

A complementary explanation could be added to account for the results of both the negative and positive context sentences in the form of a psychophysiological response that reflects the congruity between the participants' emotional state and the emotional target sentence. Indeed, High-Mood Volatility HPS participants would perceive the positive target words as being emotionally congruent with their mood, and the negative target words as being incongruent with their mood. More precisely, for the negative context sentences, we observed an enhancement of N400 in the congruent condition, that is when the target sentence was negative. In contrast, for the positive context sentences, we observed an enhancement of N400 in the incongruent condition, that is when the target sentence was also negative. Thus, for the High-Mood Volatility HPS participants, the emotional matching between their own emotional state and the target sentences would seem to be stronger than the equivalent matching between the context sentences and the target sentences. It should be noted that our results were independent of age and level of education, because our effects persisted even after removing the part of variance due to these two variables, included as covariates in our statistical analyses.

### LPC

Absence of significant results in the LPC window seems to indicate that there was no difference between the two groups (i.e. as a function of the level of hypomanic personality trait) in the late-mobilized processes involved in the integration of emotional sentences. Furthermore, our results indicated that there was no difference in the processing of the positive and negative emotional contexts in the late-emerging processes. This result therefore suggests that the emotional information was processed at a global level independently of the valence of the emotional context sentence by both the Low and High-Mood Volatility HPS group. This difference in patterns between these two components could be due to the fact that the N400 component and the LPC do not reflect the same cognitive processes. Indeed, it has been shown that the LPC reflects "an aspect of cognitive or executive control of language" [25]. Indeed, the LPC reflects the general updating of the context [19] and the attentional analysis of the global meaning of the sentence [20], whereas the N400 only reflects the detection of incongruence between a prime and a target. Thus, in the High-Mood Volatility HPS group, these observations indicate that late processes involved in emotional integration are more solidly rooted than the corresponding earlier process. They suggest that the general updating of the context permits the reliable integration of both positive and negative emotional contexts. This could also illustrate the mobilization of a compensatory process in our h-M-HPS participants which occurred between the N400 and LPC time windows during the integration of the emotional context. It should be noted that the differences between our two groups concerning age and level of education have been taken into account in our results by introducing these data as covariates in our ANCOVA.

One limitation of our research relates to the proportion of women in our sample. This could limit the generalization of our results to the HP population. Indeed, there were more women than men and meta-analyses have revealed that 80% of studies report a female advantage in emotion recognition abilities [28, 29]. However, a recent study [30] has shown that the influence of gender on the ability to recognize facial and vocal emotion emerges as of 36 years of age, whereas the mean age of our sample was 23.69 years. Moreover, this limitation is not a problem for the interpretation of our results because the proportion of men and women was the same in our two sample groups.

To summarize, the present study seems to reveal changes in the modulation of the N400 component in response to negative context sentences in the participants who scored high on the Mood Volatility subscale of the HPS. These results could reflect a difficulty in integrating negative information during sentence processing. Furthermore, these participants seem to be more sensitive to the congruency between the emotional valence of the target sentence (positive) and their own emotional mood state (positive) than to the congruency between the target sentence and the emotional context. Concerning the positive context sentences, the participants with high scores on the Mood Volatility subscale of the HPS seemed to be able to detect the incongruity between a positive context sentence and a negative target sentence better than the participants who achieved low scores on the Mood Volatility subscale of the HPS. These results could reflect a positive interpretation bias. At the same time, absence of significant results for the LPC revealed alternative findings: reflecting the general updating of the context, the results seem to reveal no difficulty in integrating negative emotional context. This could reveal the possible mobilization of a compensatory process during the late stages of emotional context integration in a population with hypomanic personality trait.

Although our study has provided interesting results, further research is needed to compare emotional context integration in bipolar population hypomanic personality trait populations. Furthermore, and regarding the question of whether emotional integration can be modulated by the participant's mood state (e.g. [31]), it would be interesting to conduct a study involving other assessments of participants' mood states.

In conclusion, our study suggested the possibility of abnormalities in the early-mobilized neurocognitive processes (N400) that mediate the processing of emotional information during the integration of emotional and contextual social information in a High-Mood Volatility HPS group.
